# Synergistic Effect of Berberine Hydrochloride and Fluconazole Against *Candida albicans* Resistant Isolates

**DOI:** 10.3389/fmicb.2020.01498

**Published:** 2020-07-02

**Authors:** Jiangyan Yong, Ruiling Zu, Xiaoxue Huang, Yiman Ge, Yan Li

**Affiliations:** ^1^Chengdu University of Traditional Chinese Medicine, Chengdu, China; ^2^Hospital of Chengdu University of Traditional Chinese Medicine, Chengdu, China; ^3^Sichuan Cancer Hospital and Institute, Chengdu, China

**Keywords:** berberine hydrochloride, fluconazole, *Candida albicans*, synergism, multiple targets

## Abstract

The emergence of resistant *Candida albicans* has made clinical fluconazole (FLC) treatment difficult. Improving sensitivity to FLC is an effective way to treat resistant isolates. Berberine hydrochloride (BBH) is a commonly used traditional Chinese medicine with antimicrobial effects, especially in resistant isolates. We investigated the molecular mechanisms underlying BBH and FLC synergism on biofilm-positive FLC-resistant *C. albicans* inhibition. Checkerboard microdilution assays and time-kill assays showed a strong synergistic effect between BBH and FLC in resistant *C. albicans* isolates, causing a significant 32–512-fold reduction in minimum inhibitory concentrations. BBH combined with FLC inhibited intracellular FLC efflux due to key efflux pump gene *CDR1* downregulation, whereas FLC alone induced high *CDR1* transcription in resistant strains. Further, BBH + FLC inhibited yeast adhesion, morphological hyphae transformation, and biofilm formation by downregulating the hyphal-specific genes *ALS3*, *HWP1*, and *ECE1*. BBH caused cytoplasmic Ca^2+^ influx, while FLC alone did not induce high intracellular Ca^2+^ levels. The vacuolar calcium channel gene *YVC1* was upregulated, while the vacuolar calcium pump gene *PMC1* was downregulated in the BBH + FLC and BBH alone groups. However, vacuolar calcium gene expression after FLC treatment was opposite in biofilm-positive FLC-resistant *C. albicans*, which might explain why BBH induces Ca^2+^ influx. These results demonstrate that BBH + FLC exerts synergistic effects to increase FLC sensitivity by regulating multiple targets in FLC-resistant *C. albicans*. These findings further show that traditional Chinese medicines have multi-target antimicrobial effects that may inhibit drug-resistant strains. This study also found that the vacuolar calcium regulation genes *YVC1* and *PMC1* are key BBH + FLC targets which increase cytoplasmic Ca^2+^ in resistant isolates, which might be critical for reversing biofilm-positive FLC-resistant *C. albicans*.

## Introduction

*Candida* is a common pathogen of nosocomial bloodstream infections, causing high-mortality invasive candidiasis. The SENTRY antifungal surveillance program showed that 46.4–57.4% of invasive candidiasis cases from 1997 to 2016 were caused by *Candida albicans* infection (Pfaller et al., 2019). Fluconazole (FLC) is a commonly used antifungal drug with a broad drug spectrum, high efficiency, and safety. However, widespread medication use has caused increased resistance annually (Xiao et al., 2018) making most FLC therapy ineffective. Thus, anti-fungal treatments face enormous challenges.

Berberine, an active component extracted from *Coptis chinensis*, which is a common traditional Chinese medicinal (TCM) herb, has a wide range of pharmacological effects and multiple-target therapeutic effects on several diseases. In particular, berberine is widely used to treat bacterial diarrhea in China. Additionally, berberine has anti-arrhythmic and anti-inflammatory activity (Lau et al., 2001; Kuo et al., 2004), reduces colorectal adenoma recurrence after polypectomy (Chen et al., 2019), decreases total cholesterol, improves insulin-resistance *in vivo*, and prevents or delays Alzheimer’s disease development associated with atherosclerosis (Cai et al., 2016; Imenshahidi and Hosseinzadeh, 2019). Furthermore, this compound exerts DNA damage-mediated antimicrobial effects on various microorganisms, including *Staphylococcus aureus*, *Pseudomonas aeruginosa*, *Escherichia coli*, *Candida albicans*, *Cryptococcus*, and *Vibrio cholerae* (Čerňáková and Košt’álová, 2002; Tillhon et al., 2012). Modern medicine indicates that Chinese herbal monomers or phytocompounds inhibit *C. albicans* growth by regulating multiple targets while inducing little drug resistance. Previous studies show that TCMs target several cellular pathways to exert antifungal effects, such as ergosterol biosynthesis suppression (Sun L. M. et al., 2015) intracellular reactive oxygen species (ROS) production (Sharma et al., 2010) inhibition of efflux pump Cdr1p and Mdr1p overexpression (Garcia-Gomes et al., 2012), biofilm inhibition (Sharma et al., 2010; Sun L. et al., 2015), and yeast apoptosis induced by intracellular or mitochondrial high Ca^2+^ levels (Yun and Lee, 2016; Tian et al., 2017). Previous studies revealed that ergosterol synthesis inhibition and apoptosis induced by endogenous ROS augmentation contribute to the synergistic effect of berberine plus FLC against *C. albicans* (Xu et al., 2009; Xu et al., 2017; Yang et al., 2018). Furthermore, this combination could also downregulate efflux pump genes *CDR1* and *CDR2* overexpression (Zhu et al., 2014).

Biofilm formation and calcium homeostasis are also important antifungal mechanisms against FLC-resistant *C. albicans*. However, there is no relevant literature exploring the synergistic antifungal effects of berberine and FLC on these two processes. Therefore, berberine hydrochloride (BBH) combined with FLC was tested to explore the molecular mechanism underlying the synergistic effect on efflux pump activity, biofilm formation, and intracellular calcium homeostasis. Synergistic molecular targets were investigated using multiple approaches to provide an effective solution for clinical treatment of drug-resistant strains.

## Materials and Methods

### Strains and Media

Fluconazole-resistant *C. albicans*, CA 0253, CA 1460, CA 2119, CA 12038, and CA 21065 (Table 1), were isolated and identified by the clinical laboratory of Chengdu University of Traditional Chinese Medicine Hospital, Chengdu, China. *C. albicans* ATCC10231 was purchased from the Guangdong Microbial Culture Collection Center Co., Ltd., China. All strains were stored in yeast extract peptone dextrose (YPD) (Hope, China) medium containing glycerol at −80°C and subcultured twice with YPD medium at 35°C for 24 h before experiments.

**TABLE 1:** Interactions of BBH with FLC against *Candida albicans*.

		MIC_80_ (μg/ml)		Interactions	
Isolate	Drugs	Alone	Combined	FICI	IN
CA 0253	FLC	512	1	0.03	SYN
	BBH	64	2		
CA 1460	FLC	>512	1	<0.06	SYN
	BBH	64	4		
CA 2119	FLC	>512	1	<0.03	SYN
	BBH	64	2		
CA 12038	FLC	>512	1	<0.03	SYN
	BBH	64	2		
CA 21065	FLC	512	1	0.03	SYN
	BBH	64	2		

**CA, C. albicans; MIC_80_, minimum inhibitory concentration inhibiting 80% C. albicans growth in the control group; FICI, fractional inhibitory concentration index; IN, Interactions; SYN, synergism*.*

### Antimicrobial Agents

Berberine hydrochloride and FLC were purchased from Chengdu Pufei De Biotech Co., Ltd., China, dissolved with dimethyl sulfoxide to achieve stock solutions of 12.8 and 20.48 mg/L, respectively, filtered using 0.22 μm filters, and stored at −20°C.

### Checkerboard Microdilution Assay

The BBH and FLC minimum inhibitory concentrations (MICs) against *C. albicans* were determined by broth microdilution assay. Drug interactions were evaluated using checkerboard microdilution assays according to CLSI (M27-A3) (CLSI, 2008). Briefly, yeast cell suspension was diluted in RPMI-1640 medium (Gibco, United States) buffered with morpholino propanesulfonic acid (MOPS) (Saiguo, China), and added to 96-well microtiter plates at a final concentration of 2 × 10^3^ CFU/mL. The serially diluted agents were subsequently added to each well. The final drug concentrations were 128–0.25 μg/mL for BBH and 32–0.5 μg/mL for FLC. Blank controls were prepared without yeast. Drug-free wells were set as growth controls. After incubation at 35°C for 24 h, prepared 2,3-bis(2-methoxy-4-nitro-5-sulfophenyl)-2H-tetrazolium-5-carboxanilide (XTT) (KeyGen, China) working solution was added to the wells and incubated in the dark for 2 h at 35°C. Finally, absorbance was measured with a microplate reader (Kehua, China) at 450 nm. MICs were defined as the lowest drug concentration inhibiting 80% *C. albicans* growth in the growth control group. The fractional inhibitory concentration index (FICI) was calculated by the following equation: FICI = MIC (A combo)/MIC (A alone) + MIC (B combo)/MIC (B alone). FICI was used to identify whether the two drugs had a synergistic antifungal effect, where FICI ≤ 0.5 indicated synergy, no synergism when FICI was between 0.5–4, and FICI ≥ 4 indicated antagonism.

### Time-Kill Curve Assay

Time-kill curve assays were performed to monitor the dynamic antifungal effect of BBH and FLC against *C. albicans* (Liu et al., 2016). The final concentrations were 2 μg/mL for BBH, 1 μg/mL for FLC, and 2 × 10^3^ CFU/mL for *C. albicans* (CA 0253). A drug-free group served as the negative control. The cells were incubated at 35°C with constant shaking (200 rpm) after different treatments. 100 μL was sampled at 0, 6, 12, 24, and 48 h in each group, and drug effects were detected with XTT tests(λ = 450 nm).

### Rh6G Efflux Assay

To evaluate the combined BBH and FLC effect on resistant *C. albicans* drug efflux, Rh6G assays were performed as previously described, with some modifications (Xu et al., 2019). The cells were first incubated with constant shaking (200 rpm) in fresh RPMI 1640 at 35°C for 2 h to exhaust cellular energy stores. A fungal suspension was added with Rh6G (Acros Organics, United States) at a final concentration of 10 μM, cultured at 35°C with constant shaking (200 rpm) for 1h, washed three times with PBS, and resuspended in PBS containing 5% glucose to 4 × 10^7^ CFU/mL. Drugs were then added, and the cells were incubated for 0, 10, 30, 60, and 120 min at 35°C in a shaker. After incubation, the supernatant was collected by centrifugation at 12,000 rpm for 1 min at room temperature. The 530 nm fluorescence of the centrifuged supernatant was detected at designated time points by a microplate reader.

### Hyphal Growth Assay

The effect of combined treatment on *C. albicans* hyphal formation was assessed using hyphal growth assays according to previous protocols, with a few modifications (Haque et al., 2016). Briefly, the cells (1 × 10^5^ CFU/mL) were treated with different drugs and incubated at 35°C with agitation (200 rpm) for 16–17 h. Unstained samples and Gram-stained samples were observed under an optical microscope and photographed (Olympus, Japan). Three random visual fields for each well and three duplicate wells for each group were also observed.

### Biofilm Information Assay

Berberine hydrochloride and FLC inhibition of *C. albicans* biofilm formation was assessed as previously described (Haque et al., 2016). Biofilm formation assays were carried out in 6-well plates incubated overnight with 10% fetal bovine serum (Tianhang, China). Cell suspensions (1 × 10^5^ CFU/mL) and drugs were added to the wells and incubated overnight at 35°C. The biofilm was washed with PBS and photographed under bright field using an inverted fluorescence microscope (Olympus, Japan) after culture (6, 12, 24, 48, and 72 h). The visual fields were photographed as described above.

### Cytoplasmic Calcium Assays

Cytoplasmic calcium assays were performed to detect intracellular calcium concentration after combination therapy (Liu et al., 2016). Briefly, overnight-cultured cells were washed and diluted with HBSS.D-Hanks buffer (Thermo Fisher, United States) (final concentration 1 × 10^7^ CFU/mL), and then mixed with 5 μM calcium indicator Fluo-3-AM (Solarbio, China) and 20% Pluronic F-127 (Meilun, China). The suspensions were incubated with agitation (200 rpm) at 35°C for 30 min, washed three times with HBSS buffer, and diluted to 1 × 10^7^ CFU/mL. After drug treatment, the cells were shaken at 35°C in the dark. Fluorescence was detected by inverted fluorescence microscopy and flow cytometry (Beckman, United States) at 0, 2, and 3 h.

### Quantitative Reverse Transcription PCR

To explore the molecular mechanism underlying the BBH and FLC synergistic effect, quantitative reverse transcription PCR (qRT-PCR) experiments were performed (Haque et al., 2016). *C. albicans* cells were cultured in YPD medium and diluted to 1 × 10^5^ CFU/mL with RPMI 1640 medium. Cells were incubated overnight with agitation (200 rpm) at 35°C with 2 μg/mL BBH and 1 μg/mL FLC. Then cells were washed and harvested for RNA extraction. Total RNA was isolated using a TRIzol RNA isolation kit (Invitrogen, United States). cDNA was synthesized using TransScript First-Strand cDNA Synthesis SuperMix (Transgen, China) for qPCR. Target gene and endogenous control (actin1) primers were designed and synthesized by Shanghai Biotech (Supplementary Table S1). The qRT-PCR reaction system was mixed with cDNA, gene primers, and TransStart Green qPCR SuperMix kit (Transgen, China) in 20 μL reaction. qRT-PCR was carried on a qTower real-time PCR system (Analytik Jena, Germany) with an initial denaturation at 94°C for 30 s, followed by 40 cycles of 94°C for 5 s, annealing at 59°C for 15 s, and extension 72°C for 10 s. Primer specificity and optimal annealing temperature were determined using melt-curve analysis. Relative target gene expression fold changes were calculated by the 2^–ΔΔ*ct*^ method.

### Statistical Analysis

Three independent experiments were performed and a drug-free group served as the negative control in all experiments. Statistical differences were analyzed by ANOVA using SPSS Statistics version 21.0 software. Data are presented as mean ± the standard error of the mean (SEM). *P* < 0.05 was considered significant.

## Results

### BBH Enhances the Susceptibility of Resistant *C. albicans* to FLC

The interactions between BBH and FLC, and treatment MICs were assessed using five *C. albicans* isolates (Table 1). The clinical isolates showed distinct biofilm formation capacity compared with biofilm-positive *C. albicans* ATCC10231. The five isolates were all FLC-resistant strains with MIC ≥ 512 μg/mL. The BBH MICs were 64 μg/mL, indicating insensitivity to both drugs. The FICI values were 0.03–0.06, indicating that BBH + FLC has strong synergistic effects. Combined use could increase *C. albicans* sensitivity to FLC and BBH, causing decreased FLC MIC from ≥512 to 1 μg/mL and reduced BBH MIC from 64 to 2–4 μg/mL. These results demonstrate that the FLC MIC is decreased by 256–512-fold with minute BBH addition. Further, these results show that BBH combined with FLC synergistically inhibits FLC-resistant *C. albicans* and significantly enhances FLC antifungal activity. Subsequent experiments were carried out with the CA 0253 strain using 2 μg/mL BBH and 1 μg/mL FLC.

**FIGURE 1:**
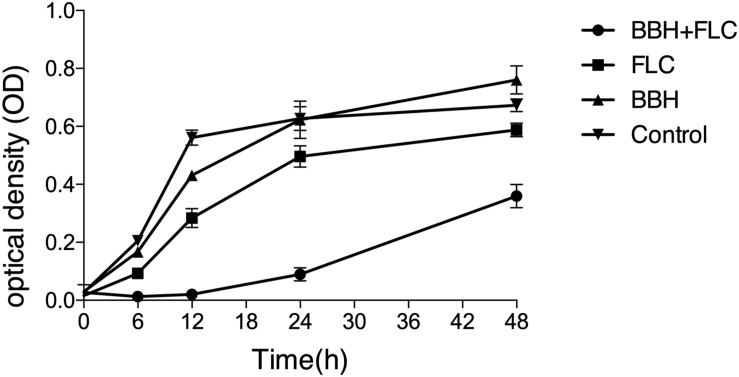
Synergistic BBH and FLC antifungal effect. CA 0253 were treated with BBH (2 μg/mL) plus FLC (1 μg/mL), FLC (1 μg/mL), BBH (2 μg/mL), and RPMI-1640 medium. The optical density (OD) was measured at 0, 6, 12, 24, and 48 h after drug treatment. Three independent experiments were performed, with eight replicates in each group (*n* = 8). Values represent means ± SEM. ANOVA tested statistical differences.

The combined BBH and FLC antifungal effect was first investigated by a 48-h dynamic time-kill study (Figure 1). Compared with the control group, growth was delayed in the other groups. However, much lower cell viability was observed in the BBH + FLC group than in the drug-monotherapy groups, especially at 0–24 h, indicating BBH + FLC treatment effectively inhibits FLC-resistant *C. albicans* growth (*p* < 0.05). A weak antifungal effect was observed in the FLC group, which was significantly lower than the combined group (*p* < 0.05). BBH alone had a poor antifungal effect, instead promoting growth at 24–48 h. These data indicate that BBH increases resistant isolate drug sensitivity, and that BBH combined with FLC synergistically inhibits *C. albicans* with a significantly dynamic antifungal effect.

### Combination of BBH and FLC Reduces Rh6G Efflux

Rh6G fluorescent substrate was used to evaluate the effect of drug combinations on drug efflux pumps. *C. albicans* actively transported the absorbed Rh6G out of the cells, indicated by gradually increased fluorescence in the supernatant over time. Compared with the control group, lower supernatant fluorescence was observed in the BBH + FLC group, FLC group, and BBH group (*p* < 0.05), showing inhibited Rh6G efflux after drug treatments (Table 2). When BBH + FLC was applied for 2 h, the extracellular Rh6G concentration was 1.43-fold lower than the FLC alone group and 1.28-fold lower than the BBH alone group (*p* < 0.05). These data indicate that BBH plus FLC significantly reduce the FLC efflux effect. Moreover, there was no significant difference between FLC or BBH treatment alone (*p* > 0.05).

**TABLE 2:** Rhodamine 6G efflux in BBH and FLC-treated *C. albicans*.

CA 0253	Time of drug action				
	0 min	10 min	30 min	60 min	2 h
BBH + FLC^◆^^★^^▲^	1069 ± 131	2472 ± 117	8596 ± 305	16892 ± 310	26019 ± 663
FLC^ 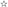 ▲^	1107 ± 169	2172 ± 101	12761 ± 69	21579 ± 982	38365 ± 1132
BBH^▲^	1466 ± 242	2946 ± 170	15366 ± 902	20047 ± 696	33294 ± 293
Control	932 ± 15	2782 ± 143	16502 ± 813	26887 ± 153	39751 ± 705

**CA 0253 were treated with BBH (2 μg/mL) plus FLC (1 μg/mL), FLC (1 μg/mL), BBH (2 μg/mL), or RPMI-1640 medium. After drug treatment, fluorescence intensity was detected (emission wavelength 530 nm) at 0, 10, 30, 60, and 120 min. Three independent experiments were performed, with eight replicates in each group (n = 8). Values represent means ± SEM. ANOVA tested statistical differences. ◆ compared with the FLC group, p < 0.05; ★ compared with the BBH group, p < 0.05; ▲compared with the control group, p < 0.05; 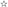 compared with the BBH group, p > 0.05*.*

### BBH Combined With FLC Inhibits Hyphae and Biofilm Formation

**FIGURE 2:**
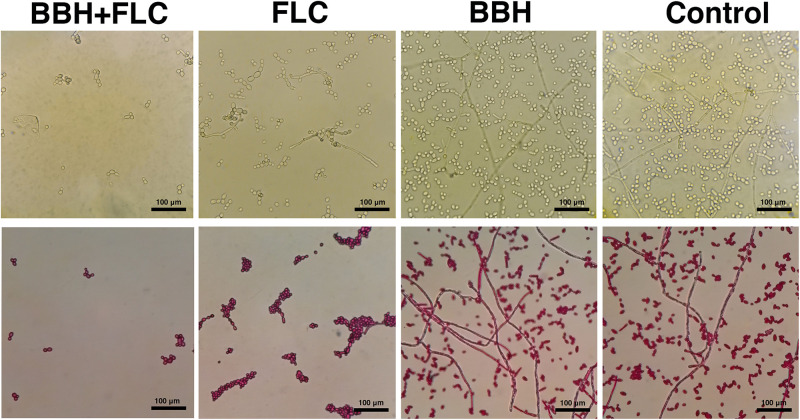
*Candida albicans* hyphae after BBH and FLC treatment. CA 0253 were treated with BBH (2 μg/mL) plus FLC (1 μg/mL), FLC (1 μg/mL), BBH (2 μg/mL), and RPMI-1640 medium. Hyphae were photographed at 40× magnification.

**FIGURE 3:**
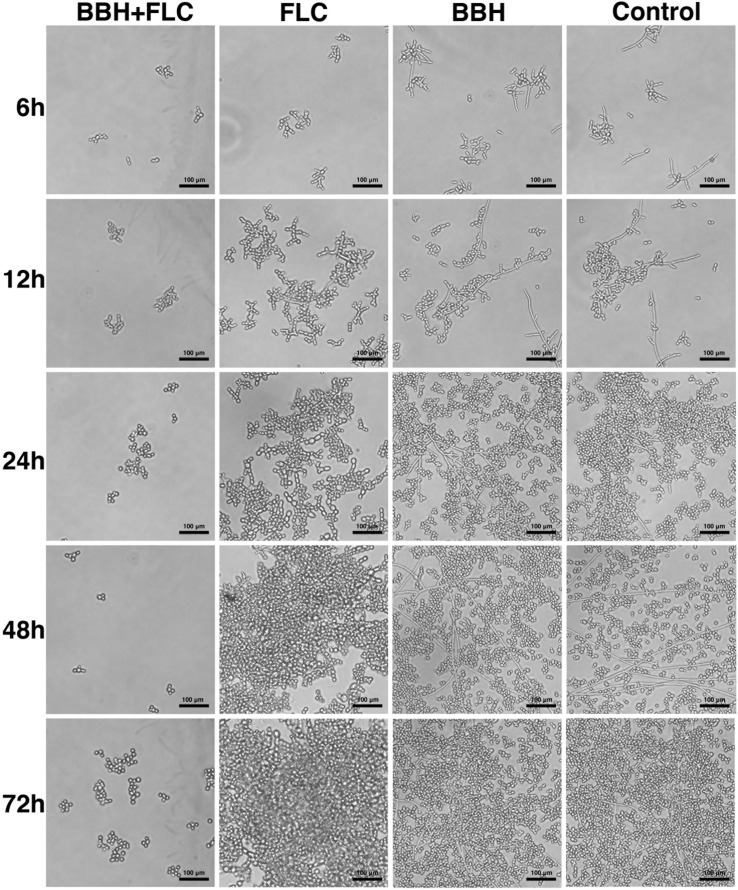
*Candida albicans* biofilm after BBH and FLC treatment. CA 0253 were treated with BBH (2 μg/mL) plus FLC (1 μg/mL), FLC (1 μg/mL), BBH (2 μg/mL), and RPMI-1640 medium. After drug treatments, biofilm was photographed (40× magnification) at 6, 12, 24, 48, and 72 h.

The biofilm-producing strain CA 0253 was used to detect the effect of BBH combined with FLC on yeast-to-hyphae conversion (Figure 2) and biofilm formation (Figure 3). Hyphal growth was absent in the presence of BBH + FLC, with very few spherical yeast cells. FLC monotherapy significantly increased the number of fungal cells, and yeast-to-hyphae conversion occurred in a portion of cells, accompanied with pseudohyphae formation. The number of fungal cells in the BBH alone group and the control group significantly increased with extensive hyphae forming a network.

Berberine hydrochloride combined with FLC completely inhibited biofilm production within 6–72 h. Only a few cells remained in the yeast form without obvious hyphae. Notably, BBH plus FLC significantly reduced yeast cell surface adherence, especially in the biofilm adhesion stage (0–12 h). Pseudohyphae growth (ellipsoidal cells joined end to end) was observed in FLC alone group, and numerous pseudohyphae formed and adhered to the surface at 24–72 h, forming an aggregated cell population. The BBH alone and the control group contained complex biofilm structure with hyphae (chains of cylindrical cells), pseudohyphae, and yeast-form cells. Hyphae growth appeared at 6 h. Hyphal cells continued to elongate at 12 h. Numerous long hyphae formed and adhered to the surface at 24–48 h, accompanied with yeast-form cells and pseudohyphae that accumulated around the hyphal cells. These data indicate that BBH combined with FLC inhibits yeast adherence and hyphae development, causing biofilm formation defects.

### BBH Plus FLC Increases Cytoplasmic Calcium

**FIGURE 4:**
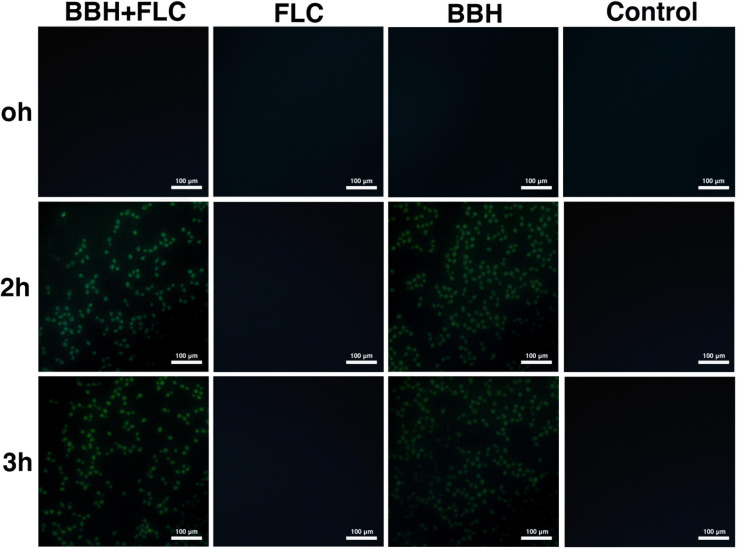
Intracellular calcium influx in *C. albicans* after BBH and FLC treatment. CA 0253 were treated with BBH (2 μg/mL) plus FLC (1 μg/mL), FLC (1 μg/mL), BBH (2 μg/mL), and RPMI-1640 medium. Cells were photographed (40× magnification) at 0, 2, and 3 h.

Inverted fluorescence microscopy was used to observe cellular calcium levels (Figure 4). The BBH plus FLC and BBH alone groups showed pale green fluorescence at 2 and 3 h, indicating Ca^2+^ influx. However, no fluorescence was observed in the FLC monotherapy or control groups, indicating no Ca^2+^ influx.

Flow cytometry was performed to compare the cytoplasmic Ca^2+^ concentration (Table 3). Compared with control and drug-monotherapy groups, higher fluorescence was observed in the BBH + FLC group at 0, 2, and 3 h (*p* < 0.05). Further, the fluorescence of BBH + FLC group at 2 h was 1. 17-, 1. 07-, and 1.18-fold higher than the FLC monotherapy, BBH alone, and control groups, respectively (*p* < 0.05). The fluorescence after BBH treatment alone was higher than after FLC alone (*p* < 0.05), but there was no significant difference between the FLC alone and control groups (*p* > 0.05). These observations indicate that BBH further increases intracellular calcium concentration, disrupting *C. albicans* calcium homeostasis.

**TABLE 3:** Intracellular Ca^2+^ fluorescence in *C. albicans* after BBH and FLC treatment.

CA 0253	Time of drug action		
	0 h	2 h	3 h
BBH + FLC^◆★^^▲^	4364.70 ± 44.33	5623.23 ± 41.16	5492.60 ± 11.00
FLC^★△^	3389.07 ± 4.18	4793.57 ± 9.49	4679.17 ± 24.69
BBH^▲^	4158.93 ± 53.24	5266.37 ± 14.98	5080.40 ± 16.00
Control	3169.30 ± 54.53	4773.53 ± 45.29	4736.17 ± 16.4

**CA 0253 were treated with BBH (2 μg/mL) plus FLC (1 μg/mL), FLC (1 μg/mL), BBH (2 μg/mL), and RPMI-1640 medium. Fluorescence was measured (emission: 530 nm) at 0, 2, and 3 h. Three independent experiments were performed, with eight replicates in each group (n = 8). Values represent means ± SEM. ANOVA tested statistical differences. ◆ compared with the FLC group, p < 0.05; ★ compared with the BBH group, p < 0.05; ▲compared with the control group, p < 0.05; Δ compared with the control group, p > 0.05*.*

### BBH Combined With FLC Induces Expression of Multiple Genes

**FIGURE 5:**
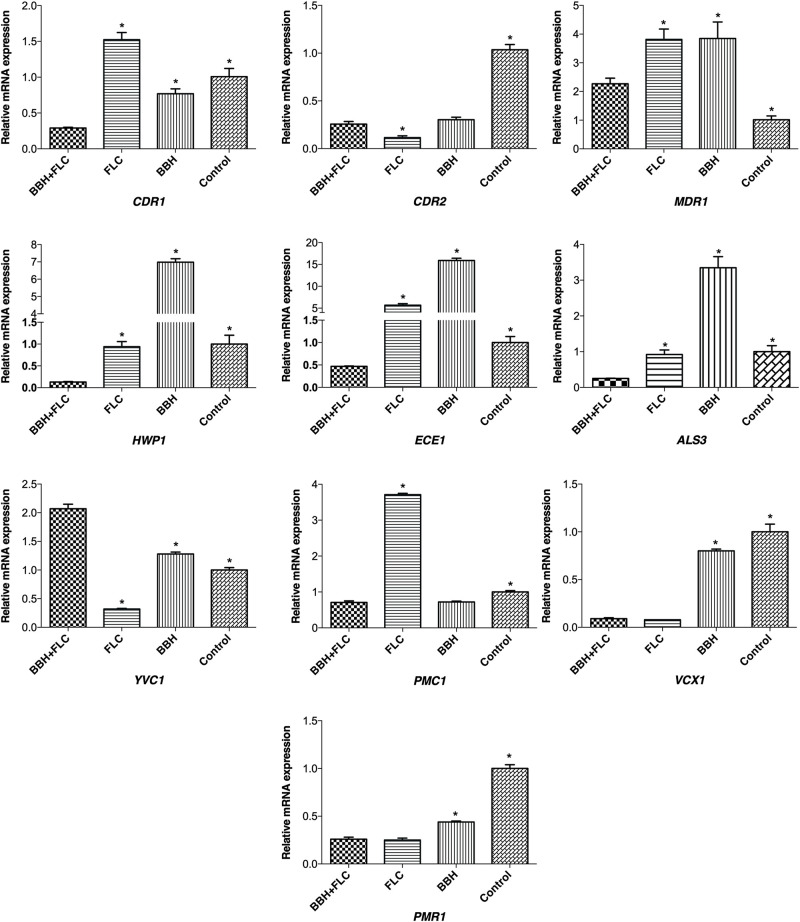
Genes expression in *C. albicans* after BBH and FLC treatment. CA 0253 were treated with BBH (2 μg/mL) plus FLC (1 μg/mL), FLC (1 μg/mL), BBH (2 μg/mL), and RPMI-1640 medium. Three independent experiments were performed, with 8 replicates in each group (*n* = 8). *p < 0.05, compared to the BBH + FLC groups, ANOVA.

qRT-PCR was conducted to explore the effect of BBH + FLC on drug-resistance, biofilm-related, and calcium-related genes (Figure 5). Compared with the control and drug-monotherapy groups, *CDR1* transcription in the BBH + FLC group was downregulated 3-to-5-fold (*p* < 0.05). However, FLC alone caused 1.52-fold *CDR1* upregulation (*p* < 0.05). Although BBH plus FLC significantly downregulated *CDR2* by 3.58-fold, much lower *CDR2* expression was observed in the FLC alone group than in the other groups (*p* < 0.05). *MDR1* expression in the combined group was almost 1.70-fold lower than in the drug-monotherapy groups (*p* < 0.05). No significant difference was detected between FLC or BBH treatment alone.

*HWP1*, *ECE1*, and *ALS3* expression in the BBH + FLC group was significantly decreased by 7. 26-, 12. 20-, and 3.73-fold, respectively, compared to FLC alone (*p* < 0.05). Further, their expression was substantially reduced by 54. 11-, 34. 20-, and 13.62-fold, respectively, compared with BBH alone (*p* < 0.05). There was no significant difference in *HWP1* (*p* = 0.499) or *ALS3* (*p* = 0.396) expression between the FLC alone and control groups, while *ECE1* expression was increased 5.68-fold after FLC treatment alone (*p* < 0.05).

Compared with the control group, FLC alone downregulated *YVC1* expression. However, *YVC1* was upregulated after BBH + FLC therapy and BBH monotherapy (*p* < 0.05). Nonetheless, *YVC1* expression in the combined group was 1.62- and 6.47-fold higher than in the BBH alone and FLC alone groups, respectively (*p* < 0.05). Compared with the control group, *PMC1* expression increased when exposed to FLC alone, and decreased when exposed to BBH + FLC or BBH alone (*p* < 0.05). BBH + FLC significantly downregulated *PMC1* by 5.28-fold compared with FLC alone (*p* < 0.05). There was no significant difference between the combined group and the BBH alone group. BBH + FLC and FLC alone significantly downregulated *VCX1* and *PMR1* expression, but the difference between the groups was not significant difference. Combined BBH and FLC significantly downregulated *VCX1* and *PMR1* expression by 8.89- and 1.69-fold, respectively, compared with BBH alone (*P* < 0.05). These results indicate that BBH combined with FLC significantly downregulates genes for the efflux pump *CDR1*, hyphal-associated *ALS3*, *HWP1*, and ECE1, and the calcium pump *PMC1*.

## Discussion

Berberine has multiple antibacterial and antifungal activities, which suppress Gram-positive and Gram-negative bacteria, and also suppress FLC-resistant *Candida* and *Cryptococcus neoformans* (Čerňáková and Košt’álová, 2002; Tillhon et al., 2012; da Silva et al., 2016). Previous studies have shown that berberine induces a significant increase in DNA strand break and DNA damage. Berberine not only destroys the cell wall integrity in *C. albicans*, but also targets the cell membrane by affecting ergosterol synthesis, resulting in increased membrane permeability (da Silva et al., 2016; Zorić et al., 2017). In our study, BBH treatment alone exerted weak antifungal effects for all resistant isolates. However, it has been reported that high doses of berberine can cause functional damage to the lungs, liver, and intestines of experimental animals. Therefore, combination therapy will be an effective strategy to reduce the toxic side effects of berberine. Because BBH + FLC will produce synergistic effect and enhance drug sensitivity, thereby significantly reducing effective drug concentration and reducing the possibility of toxic and side effects (Singh et al., 2018). Time-kill curve assays further demonstrated that the dynamic antifungal effect of combined BBH and FLC was significantly better than the drug-monotherapy groups within 48 h. Efflux pump, biofilm, and calcium-signaling pathways are important factors underlying *C. albicans* FLC resistance. Importantly, these cellular processes are not independent, but interact with each other in the fungus. Constitutive efflux pump upregulation, including CDR1, CDR2, and MDR1, is a key contributor to early biofilm resistance in *C. albicans* (Nobile and Johnson, 2015). Luna-Tapia et al. (2019) demonstrated that the calcium pump Pmc1p is essential for transformation from yeast-to-hyphae and biofilm formation. Previous work indicated that the vacuolar calcium channel Yvc1p participates in hyphal elongation and maintenance by regulating hyphal-associated gene expression (Yu et al., 2014). In this study, the effects of combined BBH and FLC on mechanisms leading to FLC resistance were assessed to investigate possible mechanisms for increasing drug sensitivity of FLC-resistant strains.

One important reason for FLC resistance in *C. albicans* is enhanced efflux pump activity (Cdr1p, Cdr2p, and Mdr1p), causing FLC to be pumped out of the cell (Cannon et al., 2009; Dhamgaye et al., 2014; Prasad and Rawal, 2014). Antifungal agents such as farnesol or clorgyline are ATP-binding cassette superfamily (ABC) and major facilitator class (MFS) transporter inhibitors, which could reverse *C. albicans* azole resistance (Holmes et al., 2012; Černáková et al., 2019). Therefore, regulating drug transporter activity would increase FLC sensitivity. According to our results, BBH + FLC, BBH alone and FLC alone reduce Rh6G excretion by decreasing *CDR1* and *CDR2* mRNA expression. Previous studies reported that Eucalyptal D (Xu et al., 2019) geraniol (Singh and Sharma, 2018) and magnolol (Sun L. M. et al., 2015) which are substrates for Cdr1p efflux pumps, exert synergistic effects by simultaneously upregulating *CDR1* and *CDR2* expression, while competitively inhibiting FLC efflux. Numerous studies suggest that synergy results from increased intracellular drug accumulation caused by downregulated efflux pump genes in FLC-resistant strains (Garcia-Gomes et al., 2012; Zhu et al., 2014; Shao et al., 2016). Although Rh6G efflux gradually increased in all groups, much lower Rh6G efflux and *CDR1* expression were detected in the BBH + FLC group, confirming previous results. In addition, *CDR1* inhibition in the BBH + FLC group was higher than *CDR2*, because the FLC-resistant strain treated with BBH + FLC revealed considerably decreased *CDR1* mRNA expression compared with the drug-monotherapy groups. However, the inhibitory effect on *CDR2* in the BBH + FLC group was not significantly superior. Previous studies showed that deleting the *CDR1* gene significantly reduces FLC resistance, while deleting *CDR2* has a relatively weak effect (Tsao et al., 2009). Based on efflux function, both Holmes et al. (2008) and Tsao et al. (2009) demonstrated that *Cdr1p* plays the most important role in inducing azole resistance. Therefore, *CDR1* mRNA expression decreased after BBH + FLC therapy, whereas *CDR1* upregulation with FLC treatment was observed in resistant strains. These results might be a crucial reason for increasing FLC sensitivity.

*Candida albicans* biofilm formation can significantly enhance antifungal drug resistance, causing increased azole MIC values by more than 1,000-fold. However, no biofilm-specific drugs exist today (Nobile and Johnson, 2015). *C. albicans* is polymorphic and capable of undergoing reversible morphological transformation between yeast, pseudohyphae, and hyphae (Sudbery et al., 2004; Noble et al., 2017). Inhibiting the yeast-to-hyphae transition can lead to biofilm formation defects, which is a new target for biofilm-specific therapeutics (Romo et al., 2017; Vila et al., 2017). We found that hyphae formation in *C. albicans* was effectively inhibited by combined BBH + FLC treatment, with very few yeast cells remaining after treatment. However, hyphae formation was not inhibited in the drug-monotherapy groups and was accompanied by numerous hyphae and pseudohyphae. The formation of hyphae upregulates the expression of the hyphal-specific genes *HWP1*, *ALS3*, and *ECE1* in the core filamentation response network, maintaining filament morphology and function (Finkel and Mitchell, 2011; Koch et al., 2018). Our results showed that BBH + FLC causes *C. albicans* hyphal structure formation failure by inhibiting *HWP1*, *ECE1*, and *ALS3* expression. The drug-monotherapy groups could not effectively inhibit hyphal-specific gene expression, such as *ECE1* upregulation after FLC exposure, or *HWP1*, *ALS3*, and *ECE1* upregulation after BBH exposure, indicated by numerous hyphae or pseudohyphae. Hyphae are physical scaffolds for yeast cell adhesion and aggregation, which enable increased biofilm strength, integrity, and maturation (Haque et al., 2016; Lee et al., 2019). *HWP1* mutants produce a thin biofilm with less hyphae *in vitro*, but display serious biofilm defects *in vivo*, only forming yeast microcolonies (Nobile et al., 2006b). *ALS3* mutants are able to form hyphae, but exhibit defects in biofilm formation (Nobile et al., 2006a, 2008). Our results support this observation. Indeed, only the combined BBH + FLC group had biofilm defects, which might be related to hyphae-specific gene inhibition. In addition, *ALS3* and *HWP1* are also capable of regulating the initial adhesion of yeast cells to surfaces, which is essential for all stages of biofilm development (Nobile et al., 2006a; Nobile and Johnson, 2015). Compared with other groups, the BBH + FLC group had significantly reduced yeast cell surface adherence, which inhibited the development of the initial basal cell layer of biofilm formation (0–12 h), thereby suppressing biofilm formation. This inhibition might be associated with downregulated *HWP1* and *ALS3* expression.

Intracellular calcium is closely related to the regulation of stress responses, antifungal drug resistance, and morphogenetic filament conversion in *C. albicans* (Juvvadi et al., 2014; Liu et al., 2015). Cytoplasmic Ca^2+^ in *C. albicans* is usually low, and calcium hypersensitivity induced by high cytoplasmic Ca^2+^ leads to toxicity and cell death (Li et al., 2018). Based on cytoplasmic calcium assay results, FLC alone failed to disrupt Ca^2+^ homeostasis in FLC-resistant *C. albicans*, but BBH + FLC and BBH monotherapy increased cytoplasmic Ca^2+^. These results indicate that BBH might be a key factor in inducing high cytoplasmic Ca^2+^. The calcium cell survival (CCS) pathway is the major calcium-signaling pathway maintaining Ca^2+^ homeostasis in *C. albicans* (Li et al., 2018). Indeed, CCS pathway activation induces calcium-related gene expression in response to increased Ca^2+^, which decreases the intracellular Ca^2+^ concentration by transporting excess cytoplasmic Ca^2+^ into internal compartments, including vacuoles, endoplasmic reticulum, and the Golgi apparatus (Juvvadi et al., 2014; Liu et al., 2016). RT-qPCR results showed that BBH + FLC and BBH monotherapy significantly upregulates *YVC1* and downregulates *PMC1*, while FLC monotherapy had the opposite effect. Vacuoles serves as the major storage site for excess Ca^2+^ in *C. albicans*. *Yvc1p* localized on the vacuolar membrane mediates Ca^2+^ release from the vacuole into the cytoplasm, while the P-type ATPase *Pmc1p* translocates Ca^2+^ from cytoplasm into the vacuole using ATP hydrolysis (Bouillet et al., 2012; Luna-Tapia et al., 2019). According these previous studies and our results, BBH + FLC and BBH monotherapy promote Ca^2+^ release from the vacuole into the cytoplasm by upregulating *YVC1* and reduce excess cytoplasmic Ca^2+^ transport into the vacuole by downregulating *PMC1*. Together, this causes increased cytoplasmic Ca^2+^, which enhances drug sensitivity in FLC-resistant *C. albicans*. However, *YVC1* could be downregulated to prevent Ca^2+^ transport into the cytoplasm after FLC treatment. In addition, upregulated *PMC1* promotes Ca^2+^ transport into the vacuole and effectively prevents increased cytoplasmic Ca^2+^, which might be an important cause of FLC resistance. The H^+^/Ca^2+^ exchanger Vcx1p transports Ca^2+^ into the vacuole using the proton-motive force across the vacuolar membrane. The calcium pump Pmr1p transfers Ca^2+^ to the Golgi apparatus (Förster and Kane, 2000; Jiang et al., 2018). In our study, both FLC monotherapy and BBH + FLC downregulated *VCX1* and *PMR1*, but there was no statistical difference. Luna-Tapia et al. (2019) reported that pmc1Δ/Δ mutants are severely impaired by high Ca^2+^ concentration in the medium, because they are unable to transport Ca^2+^ from the cytoplasm into the vacuole. However, vcx1Δ/Δ mutants are unaffected by high Ca^2+^, demonstrating that Pmc1p is required for *C. albicans* pathogenicity, FLC tolerance, and hyphal growth (Luna-Tapia et al., 2019). Thus, *YVC1* and *PMC1* might be the most important calcium-related genes to maintain cellular calcium homeostasis in FLC-resistant *C. albicans*, and may be antifungal therapy targets. The flow cytometry results showed that the cytoplasmic Ca^2+^ in the BBH + FLC group was higher than in the BBH monotherapy group, indicating that their combined use further enhances cytoplasmic Ca^2+^. Although there was no significant difference in *PMC1* expression, *YVC1* expression in the BBH + FLC group was higher than in the BBH monotherapy group, which might explain the higher cytoplasmic Ca^2+^ in the BBH + FLC group.

Our study found that BBH + FLC treatment exerts a synergistic antifungal effect by regulating efflux pumps, hyphae, and calcium-related pathways. One limitation of this study is that additional synergistic regulatory sites need to be further explored. Hyphae are a key factor for *C. albicans* virulence and invasiveness, and some researchers observed that Ca^2+^-regulated genes *YVC1* and *PMC1* deletion cause hyphae defects in *C. albicans* (Yu et al., 2014; Luna-Tapia et al., 2019). We found that combined BBH + FLC simultaneously regulates vacuolar Ca^2+^-regulated genes and significantly inhibits yeast-to-hyphae conversion. Therefore, how BBH + FLC modulates vacuolar Ca^2+^ regulation and hyphae formation in biofilm-positive FLC-resistant *C. albicans* will be explored in future studies. We also found that the Ca^2+^ channel, *YVC1*, and the Ca^2+^ pump, *PMC1*, increase cytoplasmic Ca^2+^ in *C. albicans*, and gene transcription level of resistant isolate treated with BBH + FLC and FLC alone were completely opposite. This finding informs further study of key targets to inhibit biofilm-positive FLC-resistant *C. albicans*.

## Conclusion

Berberine hydrochloride synergistically suppresses FLC efflux, hyphae and biofilm formation, and induces high cytoplasmic Ca^2+^, indicating that the combination could restore FLC antifungal activity in FLC-resistant *C. albicans* by regulating multiple targets. This paper provides state-of-the-art TCM antimicrobial research, demonstrates that TCMs have multi-target antimicrobial effects, and suggests new ideas for resistant strain treatments. These findings clearly suggest that BBH + FLC may be an effective therapeutic option for infections related to FLC-resistant *C. albicans*, especially biofilm-positive resistant isolates. Future experiments will explore the relationship between hyphae formation and Ca^2+^ signaling pathways, and further study the key nodes inhibiting biofilm-positive FLC-resistant *C. albicans*.

## Data Availability Statement

The datasets generated for this study are available on request to the corresponding author.

## Ethics Statement

This study was carried out in accordance with the recommendations of Specimen Collection and Transport in Clinical Microbiology (WS/T640-2018), People’s Republic of China Health Industry Standard. The protocol was approved by the National Health Commission of the People’s Republic of China. Informed consent was not needed as this study was retrospective without involving any information from patients.

## Author Contributions

JY and YL conceived and designed the experiments. JY performed the experiments. JY, RZ, XH, YG, and YL contributed to reagents, data analysis, and interpretation. JY and YL wrote the manuscript. All authors approved the manuscript for publication.

## Conflict of Interest

The authors declare that the research was conducted in the absence of any commercial or financial relationships that could be construed as a potential conflict of interest.

## Supplementary Material

TABLE S1 Primer sequences used in this study.

## References

[B1] Bouillet L. Cardoso A. S. Perovano E. Pereira R. Ribeiro E. M. Trópia M. J. M. (2012). The involvement of calcium carriers and of the vacuole in the glucose-induced calcium signaling and activation of the plasma membrane H+-ATPase in *Saccharomyces cerevisiae* cells. *Cell Calcium* 51 72–81. 10.1016/j.ceca.2011.10.008 22153127

[B2] Cai Z. Wang C. Yang W. (2016). Role of berberine in Alzheimer’s disease. *Neuropsychiatr. Dis. Treat.* 12:2509. 10.2147/ndt.s114846 27757035 PMC5055107

[B3] Cannon R. D. Lamping E. Holmes A. R. Niimi K. Baret P. V. Keniya M. V. (2009). Efflux-mediated antifungal drug resistance. *Clin. Microbiol. Rev.* 22 291–321. 19366916 10.1128/CMR.00051-08 PMC2668233

[B4] Černáková L. Dižová S. Gášková D. JanČíková I. Bujdáková H. (2019). Impact of farnesol as a modulator of efflux pumps in a fluconazole-resistant strain of *Candida albicans*. *Microb. Drug Resist.* 25 805–812. 10.1089/mdr.2017.0332 30785845

[B5] Čerňáková M. Košt’álová D. (2002). Antimicrobial activity of berberine—A constituent of *Mahonia aquifolium*. *Folia Microbiol.* 47 375–378. 10.1007/bf02818693 12422513

[B6] Chen Y.-X. Gao Q.-Y. Zou T.-H. Wang B.-M. Liu S.-D. Sheng J.-Q. (2019). *Berberine Hydrochloride For The Prevention Of Colorectal Adenomas: A Double-Blind, Randomised Controlled Multicenter Clinical Trial.* Available online at: https://ssrn.com/abstract=3414428

[B7] CLSI (2008). *Reference Methods for Broth Dilution Antifungal Susceptibility Testing Of Yeast; Approved Standard-Third Edition, CLSI Document M27-A3.* Wayne, PA: CLSI.

[B8] da Silva A. R. de Andrade Neto J. B. da Silva C. R. de Sousa Campos R. Silva R. A. C. Freitas D. D. (2016). Berberine antifungal activity in fluconazole-resistant pathogenic yeasts: action mechanism evaluated by flow cytometry and biofilm growth inhibition in *Candida* spp. *Antimicrob. Agents Chemother.* 60 3551–3557. 10.1128/aac.01846-15 27021328 PMC4879420

[B9] Dhamgaye S. Devaux F. Vandeputte P. Khandelwal N. K. Sanglard D. Mukhopadhyay G. (2014). Molecular mechanisms of action of herbal antifungal alkaloid berberine, in *Candida albicans*. *PLoS One* 9:e104554. 10.1371/journal.pone.0104554 25105295 PMC4126717

[B10] Finkel J. S. Mitchell A. P. (2011). Genetic control of *Candida albicans* biofilm development. *Nat. Rev. Microbiol.* 9:109. 10.1038/nrmicro2475 21189476 PMC3891587

[B11] Förster C. Kane P. M. (2000). Cytosolic Ca2+ homeostasis is a constitutive function of the V-ATPase in *Saccharomyces cerevisiae*. *J. Biol. Chem.* 275 38245–38253. 10.1074/jbc.m006650200 10991947

[B12] Garcia-Gomes A. Curvelo J. Soares R. A. Ferreira-Pereira A. (2012). Curcumin acts synergistically with fluconazole to sensitize a clinical isolate of *Candida albicans* showing a MDR phenotype. *Med. Mycol.* 50 26–32. 10.3109/13693786.2011.578156 21539505

[B13] Haque F. Alfatah M. Ganesan K. Bhattacharyya M. S. (2016). Inhibitory effect of sophorolipid on *Candida albicans* biofilm formation and hyphal growth. *Sci. Rep.* 6:23575. 10.1038/srep23575 PMC4876995 27030404

[B14] Holmes A. R. Keniya M. V. Ivnitski-Steele I. Monk B. C. Lamping E. Sklar L. A. (2012). The monoamine oxidase A inhibitor clorgyline is a broad-spectrum inhibitor of fungal ABC and MFS transporter efflux pump activities which reverses the azole resistance of *Candida albicans* and Candida glabrata clinical isolates. *Antimicrob. Agents Chemother.* 56 1508–1515. 10.1128/aac.05706-11 22203607 PMC3294898

[B15] Holmes A. R. Lin Y.-H. Niimi K. Lamping E. Keniya M. Niimi M. (2008). ABC transporter Cdr1p contributes more than Cdr2p does to fluconazole efflux in fluconazole-resistant *Candida albicans* clinical isolates. *Antimicrob. Agents Chemother.* 52 3851–3862. 10.1128/aac.00463-08 18710914 PMC2573144

[B16] Imenshahidi M. Hosseinzadeh H. (2019). Berberine and barberry (Berberis vulgaris): a clinical review. *Phytother. Res.* 33 504–523. 10.1002/ptr.6252 30637820

[B17] Jiang L. Wang J. Asghar F. Snyder N. Cunningham K. W. (2018). CaGdt1 plays a compensatory role for the calcium pump CaPmr1 in the regulation of calcium signaling and cell wall integrity signaling in *Candida albicans*. *Cell Commun. Signal.* 16:33. 10.1186/s12964-018-0246-x PMC6025805 29954393

[B18] Juvvadi P. R. Lamoth F. Steinbach W. J. (2014). Calcineurin as a multifunctional regulator: unraveling novel functions in fungal stress responses, hyphal growth, drug resistance, and pathogenesis. *Fungal Biol. Rev.* 28 56–69. 10.1016/j.fbr.2014.02.004 25383089 PMC4219591

[B19] Koch B. Barugahare A. A. Lo T. L. Huang C. Schittenhelm R. B. Powell D. R. (2018). A metabolic checkpoint for the yeast-to-hyphae developmental switch regulated by endogenous nitric oxide signaling. *Cell Rep.* 25 2244–2258. 30463019 10.1016/j.celrep.2018.10.080

[B20] Kuo C.-L. Chi C.-W. Liu T.-Y. (2004). The anti-inflammatory potential of berberine in vitro and in vivo. *Cancer Lett.* 203 127–137. 10.1016/j.canlet.2003.09.002 14732220

[B21] Lau C. W. Yao X. Q. Chen Z. Y. Ko W. H. Huang Y. (2001). Cardiovascular actions of berberine. *Cardiovasc. Drug. Rev.* 19 234–244. 10.1111/j.1527-3466.2001.tb00068.x 11607041

[B22] Lee J.-H. Kim Y.-G. Khadke S. K. Yamano A. Watanabe A. Lee J. (2019). Inhibition of biofilm formation by *Candida albicans* and polymicrobial microorganisms by nepodin via hyphal-growth suppression. *ACS Infect. Dis.* 5 1177–1187. 10.1021/acsinfecdis.9b00033 31055910

[B23] Li Y. Sun L. Lu C. Gong Y. Li M. Sun S. (2018). Promising antifungal targets against *Candida albicans* based on ion homeostasis. *Front. Cell. Infect. Microbiol.* 8:286. 10.3389/fcimb.2018.00286 30234023 PMC6131588

[B24] Liu S. Hou Y. Liu W. Lu C. Wang W. Sun S. (2015). Components of the calcium-calcineurin signaling pathway in fungal cells and their potential as antifungal targets. *Eukaryot. Cell* 14 324–334. 10.1128/ec.00271-14 25636321 PMC4385803

[B25] Liu S. Yue L. Gu W. Li X. Zhang L. Sun S. (2016). Synergistic effect of fluconazole and calcium channel blockers against resistant *Candida albicans*. *PLoS One* 11:e0150859. 10.1371/journal.pone.0150859 26986478 PMC4795682

[B26] Luna-Tapia A. DeJarnette C. Sansevere E. Reitler P. Butts A. Hevener K. E. (2019). The vacuolar Ca2+ ATPase Pump Pmc1p is required for *Candida albicans* pathogenesis. *mSphere* 4:e00715-18. 10.1128/mSphere.00715-18 PMC6365616 30728284

[B27] Nobile C. J. Johnson A. D. (2015). *Candida albicans* biofilms and human disease. *Annu. Rev. Microbiol.* 69 71–92. 26488273 10.1146/annurev-micro-091014-104330 PMC4930275

[B28] Nobile C. J. Andes D. R. Nett J. E. Smith F. J. Jr. Yue F. Phan Q.-T. (2006a). Critical role of Bcr1-dependent adhesins in *C. albicans* biofilm formation in vitro and in vivo. *PLoS Pathog.* 2:e63. 10.1371/journal.pone.0000063 16839200 PMC1487173

[B29] Nobile C. J. Nett J. E. Andes D. R. Mitchell A. P. (2006b). Function of *Candida albicans* adhesin Hwp1 in biofilm formation. *Eukaryot. Cell* 5 1604–1610. 10.1128/ec.00194-06 17030992 PMC1595337

[B30] Nobile C. J. Schneider H. A. Nett J. E. Sheppard D. C. Filler S. G. Andes D. R. (2008). Complementary adhesin function in *C. albicans* biofilm formation. *Curr. Biol.* 18 1017–1024. 10.1016/j.cub.2008.06.034 18635358 PMC2504253

[B31] Noble S. M. Gianetti B. A. Witchley J. N. (2017). *Candida albicans* cell-type switching and functional plasticity in the mammalian host. *Nat. Rev. Microbiol.* 15:96. 10.1038/nrmicro.2016.157 27867199 PMC5957277

[B32] Pfaller M. A. Diekema D. J. Turnidge J. D. Castanheira M. Jones R. N. (2019). Twenty years of the SENTRY antifungal surveillance program: results for *Candida* species from 1997–2016. *Open Forum Infect. Dis.* 6 S79–S94. 30895218 10.1093/ofid/ofy358 PMC6419901

[B33] Prasad R. Rawal M. K. (2014). Efflux pump proteins in antifungal resistance. *Front. Pharmacol.* 5:202. 10.3389/fcimb.2018.00202 25221515 PMC4148622

[B34] Romo J. A. Pierce C. G. Chaturvedi A. K. Lazzell A. L. McHardy S. F. Saville S. P. (2017). Development of anti-virulence approaches for candidiasis via a novel series of small-molecule inhibitors of *Candida albicans* filamentation. *mBio* 8:e01991-17. 10.1128/mBio.01991-17 PMC5717394 29208749

[B35] Shao J. Zhang M. Wang T. Li Y. Wang C. (2016). The roles of CDR1, CDR2, and MDR1 in kaempferol-induced suppression with fluconazole-resistant *Candida albicans*. *Pharm. Biol.* 54 984–992. 10.3109/13880209.2015.1091483 26459663 PMC11132302

[B36] Sharma M. Manoharlal R. Puri N. Prasad R. (2010). Antifungal curcumin induces reactive oxygen species and triggers an early apoptosis but prevents hyphae development by targeting the global repressor TUP1 in *Candida albicans*. *Biosci. Rep.* 30 391–404. 10.1042/bsr20090151 20017731

[B37] Singh N. Sharma B. (2018). Toxicological Effects of berberine and sanguinarine. *Front. Mol. Biosci.* 5:21 10.3389/fcimb.2018.0021 PMC5867333 29616225

[B38] Singh S. Fatima Z. Ahmad K. Hameed S. (2018). Fungicidal action of geraniol against *Candida albicans* is potentiated by abrogated CaCdr1p drug efflux and fluconazole synergism. *PLoS One* 13:e0203079. 10.1371/journal.pone.0203079 30157240 PMC6114893

[B39] Sudbery P. Gow N. Berman J. (2004). The distinct morphogenic states of *Candida albicans*. *Trends Microbiol.* 12 317–324. 10.1016/j.tim.2004.05.008 15223059

[B40] Sun L. Liao K. Wang D. (2015). Effects of magnolol and honokiol on adhesion, yeast-hyphal transition, and formation of biofilm by *Candida albicans*. *PLoS One* 10:e0117695. 10.1371/journal.pone.0117695 25710475 PMC4339376

[B41] Sun L. M. Liao K. Liang S. Yu P. H. Wang D. Y. (2015). Synergistic activity of magnolol with azoles and its possible antifungal mechanism against *Candida albicans*. *J. Appl. Microbiol.* 118 826–838. 10.1111/jam.12737 25641229

[B42] Tian H. Qu S. Wang Y. Lu Z. Zhang M. Gan Y. (2017). Calcium and oxidative stress mediate perillaldehyde-induced apoptosis in *Candida albicans*. *Appl. Microbiol. Biotechnol.* 101 3335–3345. 10.1007/s00253-017-8146-3 28224196

[B43] Tillhon M. Ortiz L. M. G. Lombardi P. Scovassi A. I. (2012). Berberine: new perspectives for old remedies. *Biochem. Pharmacol.* 84 1260–1267. 10.1016/j.bcp.2012.07.018 22842630

[B44] Tsao S. Rahkhoodaee F. Raymond M. (2009). Relative contributions of the *Candida albicans* ABC transporters Cdr1p and Cdr2p to clinical azole resistance. *Antimicrob. Agents Chemother.* 53 1344–1352. 10.1128/aac.00926-08 19223631 PMC2663127

[B45] Vila T. Romo J. A. Pierce C. G. McHardy S. F. Saville S. P. Lopez-Ribot J. L. (2017). Targeting *Candida albicans* filamentation for antifungal drug development. *Virulence* 8 150–158. 10.1080/21505594.2016.1197444 27268130 PMC5354165

[B46] Xiao M. Sun Z.-Y. Kang M. Guo D.-W. Liao K. Chen S. C.-A. (2018). Five-year national surveillance of invasive candidiasis: species distribution and azole susceptibility from the China Hospital Invasive Fungal Surveillance Net (CHIF-NET) study. *J. Clin. Microbiol.* 56:e0577-18. 10.1128/JCM.00577-18 PMC6018329 29743305

[B47] Xu J. Liu R. Sun F. An L. Shang Z. Kong L. (2019). Eucalyptal D enhances the antifungal effect of fluconazole on fluconazole-resistant *Candida albicans* by competitively inhibiting efflux pump. *Front. Cell. Infect. Microbiol.* 9:211. 10.3389/fcimb.2018.00211 31281800 PMC6595430

[B48] Xu Y. Quan H. Wang Y. Zhong H. Sun J. Xu J. (2017). Requirement for ergosterol in berberine tolerance underlies synergism of fluconazole and berberine against fluconazole-resistant *Candida albicans* isolates. *Front. Cell. Infect. Microbiol.* 7:491 10.3389/fcimb.2018.00491 PMC5712545 29238700

[B49] Xu Y. Wang Y. Yan L. Liang R.-M. Dai B.-D. Tang R.-J. (2009). Proteomic analysis reveals a synergistic mechanism of fluconazole and berberine against fluconazole-resistant *Candida albicans*: endogenous ROS augmentation. *J. Proteome Res.* 8 5296–5304. 10.1021/pr9005074 19754040

[B50] Yang Z. Wang Q. Ma K. Shi P. Liu W. Huang Z. (2018). Fluconazole inhibits cellular ergosterol synthesis to confer synergism with berberine against yeast cells. *J. Glob. Antimicrob. Resist.* 13 125–130. 10.1016/j.jgar.2017.12.011 29287714

[B51] Yu Q. Wang F. Zhao Q. Chen J. Zhang B. Ding X. (2014). A novel role of the vacuolar calcium channel Yvc1 in stress response, morphogenesis and pathogenicity of *Candida albicans*. *Int. J. Med. Microbiol.* 304 339–350. 10.1016/j.ijmm.2013.11.022 24368068

[B52] Yun D. G. Lee D. G. (2016). Silibinin triggers yeast apoptosis related to mitochondrial Ca2+ influx in *Candida albicans*. *Intern. J. Biochem. Cell Biol.* 80 1–9. 10.1016/j.biocel.2016.09.008 27639679

[B53] Zhu S.-L. Yan L. Zhang Y.-X. Jiang Z.-H. Gao P.-H. Qiu Y. (2014). Berberine inhibits fluphenazine-induced up-regulation of CDR1 in *Candida albicans*. *Biol. Pharm. Bull.* 37 268–273. 10.1248/bpb.b13-00734 24492724

[B54] Zorić N. Kosalec I. Tomić S. Bobnjarić I. Jug M. Vlainić T. (2017). Membrane of *Candida albicans* as a target of berberine. *BMC Complement. Altern. Med.* 17:268. 10.1186/s12906-017-1773-5 28514949 PMC5436450

