# Supplementary material for: Synergistic Effect of Berberine Hydrochloride and Fluconazole Against Candida albicans Resistant Isolates
Source: Front Microbiol. 2020 Jul 2;11:1498. doi: 10.3389/fmicb.2020.01498 (PMC7343717; doi:10.3389/fmicb.2020.01498)
Supplement: TABLE S1 — Primer sequences used in this study. [file Table_1.DOCX]

**Supplementary Table 1.** Primer sequences used in this study

| **Gene** | **Primer sequences（5’→3’）** |
| --- | --- |
| *actin1* | F: ACCACCGGTATTGTTTTGGA |
|  | R: TGGACAAATGGTTGGTCAAG |
| *CDR1* | F: GGTGCTGCCATGTTCTTTGC |
|  | R: AGGCATCAGCTGAAGGACGA |
| *CDR2* | F: ATCTACTCCTGGAAGCAC |
|  | R: GCATAGCACCTTTATTGA |
| *MDR1* | F: GCTGCTACTACTGCTTCTGGTG |
|  | R: TGAAACCCAACACGGAACTAC |
| *HWP1* | F: GCTGGTCCAGGTGCTTCTTCTTC |
|  | R: TTGGCAGATGGTTGCATGAGTGG |
| *ECE1* | F: GCCATCATCCACCATGCTCCAG |
|  | R: CAGGAACAGTAGGTGCTTGGTCAG |
| *ALS3* | F: TGTGCTGGTGGTTATTGGCA |
|  | R: AAGGTGGTCACAGCGGTAGT |
| *YVC1* | F: ATGTGCCTCTCCGTTAATGTGGTC |
|  | R: TGCGTGGCTTCGTTCTTACCATC |
| *VCX1* | F: GTGCCGATGCGATCTTGAATGAAC |
|  | R: ACAATACGACCAAGCCAGCAGTC |
| *PMC1* | F: ACGCCGTTATTACCGCTGTG |
|  | R: CAATGGCAGCAAGGAACCCA |
| *PMR1* | F: ACTGCTAGAGACACGACCATGACC |
|  | R: TGGAATAATGACGGCACGACAAGG |
